# A three-dimensional model of aortic dissection for hybrid surgical simulation and training

**DOI:** 10.1093/icvts/ivaf044

**Published:** 2025-02-27

**Authors:** Mamoru Arakawa, Koji Kawahito

**Affiliations:** Department of Cardiovascular Surgery, Jichi Medical University, Tochigi, Japan; Department of Cardiovascular Surgery, Jichi Medical University, Tochigi, Japan

**Keywords:** aortic dissection, simulation, thoracic endovascular aortic repair

## Abstract

Thoracic endovascular aortic repair is widely performed in complicated and uncomplicated type B aortic dissection cases. After the introduction of a stent graft, the use of several types of hybrid approaches has been reported for patients with type A aortic dissection. The procedure is advanced because the complications are fatal; therefore, training is required. However, the surgical simulation of aortic dissection is challenging because no favourable animal model is available. This study aimed to simulate hybrid surgical simulation using a novel three-dimensional aortic dissection model. Three-dimensional polyurethane models of type A and B aortic dissection, both of which have true and false lumens, were manufactured based on computed tomography data. Under fluoroscopy, the entry tear and false lumen flows were visualized using a contrast medium. A stent graft was delivered and deployed under pulsatile conditions in the type B aortic dissection model. Total arch replacement was performed in the type A and B aortic dissection models after thoracic endovascular aortic repair as a hybrid approach. In conclusion, a model with a mock circuit is a useful tool to simulate both open and endovascular aortic repair for aortic dissections as a hybrid approach.

## INTRODUCTION

Thoracic endovascular aortic repair (TEVAR) was initially performed to treat thoracic aortic aneurysms. The indication was later expanded to type B aortic dissection (TBAD) over the past decade. Regarding acute TBAD, the Study of Thoracic Aortic Type B Dissection Using Endoluminal Repair (STABLE) showed the advantage of TEVAR for complicated TBAD [1, 2]. The Investigation of Stent Grafts in Aortic Dissection (INSTEAD) and INSTEAD-XL studies have also shown the possible prevention of aortic events using pre-emptive TEVAR [3, 4]. Therefore, TEVAR for TBAD has become the standard therapy for complicated and uncomplicated cases. However, it is an advanced procedure because the flap and aorta are fragile, and complications such as retrograde type A aortic dissection (RTAD) are life-threatening problems [5]. In Japan, the certification of a practicing surgeon by the Japanese Committee for Stent-graft Management (JACSM) is required for performing stent procedures. Particularly, the JACSM certification of the supervising surgeons is necessary to perform stent procedures for aortic dissection. However, no practical training program is available for TEVAR in aortic dissection due to the lack of animal models for endovascular treatment. Previously, we developed a three-dimensional (3D) polyurethane model of type A aortic dissection (TAAD) for device development [6]. In this study, the model was adopted for both open and endovascular procedures to be used in simulations and training. The features of this model are feasible for both open and endovascular aortic repairs. A model with a mock circuit is a useful tool for hybrid surgical simulation as well as simple conventional aortic surgical training.

## MATERIALS AND METHODS

### Ethics approval

This study was approved by the Ethics Committee of our institute (No. 23-121). In accordance with the national guidelines of Japan, and consent was obtained through an opt-out procedure.

### Three-dimensional polyurethane aortic model

**Figure 1:**
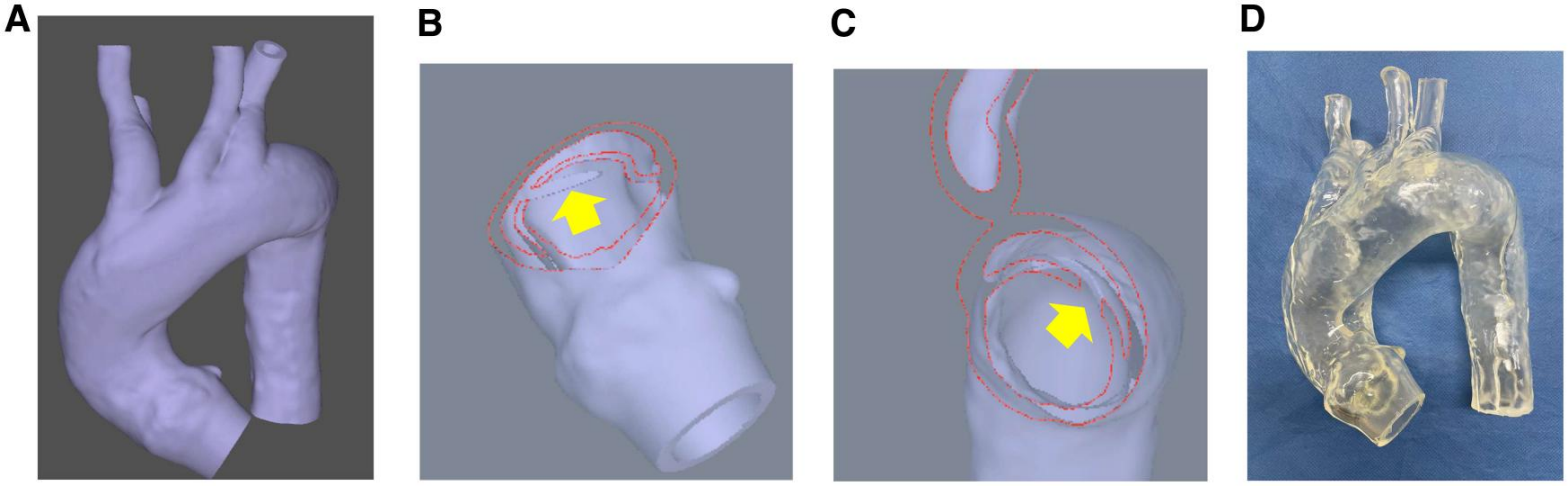
Manufacturing process of a three-dimensional (3D) polyurethane aortic dissection model. (**A**) Based on the 3D PDF from an actual patient’s images of computed tomography, the 3D soft model was manufactured. (**B**) An entry tear of 2 cm was located at the ascending aorta vertically in the type A aortic dissection model. (**C**) An entry tear of 2 cm was located at the descending aorta vertically. (**D**) The outer shapes of type A and B models are similar

Based on Digital Imaging and Communication in Medicine (DICOM) data from computed tomography of a patient with aortic dissection, we requested that Cross Medical Co. Ltd, Kyoto, Japan, create a 3D model. The manufacturing process has been described previously [6]. First, the patient’s DICOM data were imported, edited into 3D data, and exported as standard triangulated language files. The standard triangulated language files were imported and modified for model creation. Subsequently, the modified standard triangulated language data were exported to create a 3D aortic model. Both TAAD and TBAD models were developed for endovascular and surgical training (Fig. 1A). The TAAD model had an entry tear of 2 cm, located at the ascending aorta vertically at a distance of 2 cm from the sinotubular junction (Fig. 1B). In contrast, the TBAD model had an entry tear of 2 cm, located at the descending aorta vertically at a distance of 2 cm from the left subclavian artery (Fig. 1C). The outer appearances of both models were identical (Fig. 1D).

### Clinical scenarios

We considered two clinical scenarios from tear entry malperfusion classification according to the European Association for Cardio-Thoracic Surgery/Society of Thoracic Surgeons guidelines [7]. The first scenario was a conventional total arch replacement for the TAAD model (type A E1M0). Next, the TAAD model with the stent graft was fixed in an acrylic box with a sternal opener for surgical training. The total arch replacement was performed using Triplex Advanced (Terumo Corp., Tokyo, Japan) with four branches for aortic arch replacement. Second, TEVAR was performed in patients with TBAD (type B E3M0). The model was connected to a pulsatile mock circuit (AlphaFlow EC-8; Fuyo Corp., Tokyo, Japan), which can measure pressure and flow. Subsequently, the acryl box was filled with water, and the DrySheal Flex sheath (W. L. Gore and Associates, Flagstaff, Ariz) was used to prevent water leakage. A guidewire was inserted through the access route under fluorescence and was subsequently exchanged for a stiff wire. Relay Pro (Terumo Corp., Tokyo, Japan) was used, and an imaging pigtail catheter was inserted into the right brachiocephalic artery. The stent graft was deployed under fluorescence in zone 3. Subsequently, the water in the acrylic box was removed, and a sternal opener was used to perform hybrid arch replacement using the TBAD model.

## RESULTS

**Figure 2:**
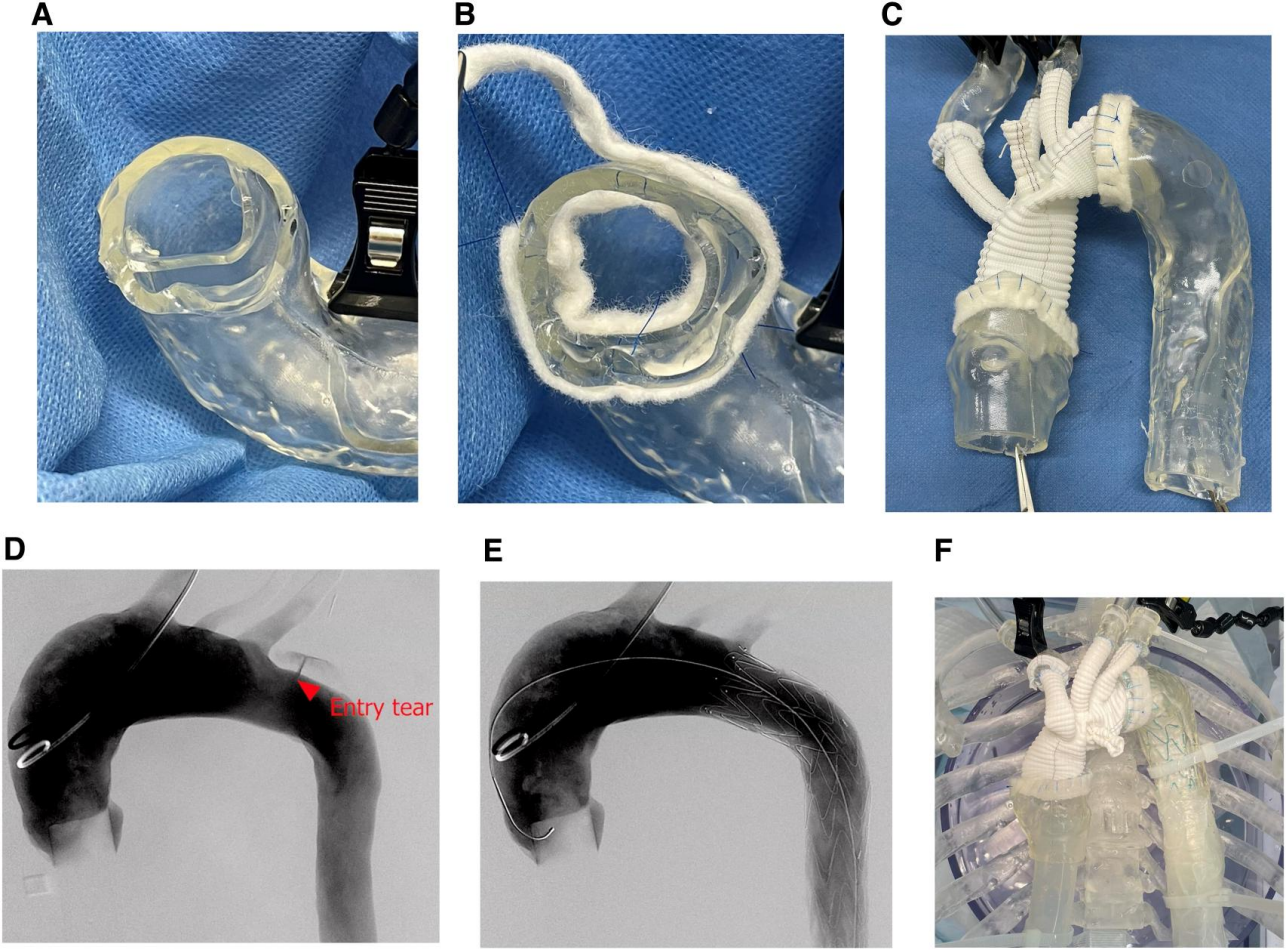
The images during both endovascular and surgical simulation. Images of the double-felt strip reinforcement during conventional total arch replacement (**A** and **B**). Model after procedure (**C**). Angiography before thoracic endovascular aortic repair (**D**), after device deployment (**E**) and after total arch replacement (**F**)

Conventional total arch replacement was successfully performed using the TAAD model. Notably, the felt strip reinforcement used to handle the dual-dissected lumens was well reproduced (Fig. 2A–C). Simulated TEVAR was successfully performed using fluoroscopy. Angiography revealed an entry tear (Fig. 2D). A delivery sheath was inserted, and the hard sheath of the Relay Pro was advanced into the descending aorta. Subsequently, the soft sheath was advanced into the aortic arch. The stent graft was deployed under pulsatile conditions (heart rate, 60 bpm; pump flow, 2.3 l/min; and pressure, 85/73 mmHg). Angiography performed after deployment revealed a type IA endoleak through the entry tear (Fig. 2E). The TEVAR procedure is shown in Video 1. Second, the 3D model with the stent graft was incised at the ascending aorta to trim the distal anastomosis site. Subsequently, the distal anastomosis site, including the proximal edge of the stent graft, was reinforced using a felt strip. The proximal anastomosis was performed with felt strip enforcement (Fig. 2F) and three-branch reconstruction using running sutures.

## DISCUSSION

In this study, we developed a dissected soft 3D aortic model for hybrid aortic surgical simulation. Both the endovascular and surgical procedures were well reproduced using this model. The model can be connected to a mock circulation for TEVAR under pulsatile conditions and subsequently anastomosed to a prosthetic graft. Medical equipment is constantly advancing; therefore, surgical procedures, particularly in cardiovascular surgery, require specific training. Several studies have demonstrated the significance of improvements in 3D printing [8]. The 3D-printed model did not reproduce the soft-dissected dual-lumen model because the resin was overly rigid to mimic the dissected aorta. However, photocopy technology allows for the creation of a more adaptable model. We can arrange the model for various situations, such as entry tear location and size, the ratio of the true and false lumens, the angle of the arch and dissection types.

Advanced TEVAR procedures, such as fenestrated and branched TEVAR, are currently being developed [9]. This model facilitates training for advanced TEVAR using various approach vessels. With this model, open surgical repair can be simulated using both conventional and hybrid approaches. Training in open and endovascular procedures is essential not only for various elective hybrid aortic surgeries [10] but also for managing possible catastrophic complications of TEVAR, including RTAD. The advantage of this model is that it is highly versatile. Because the model was transparent, endovascular procedures were simulated visually [6] and under fluoroscopy.

However, this model has some limitations. First, a model was constructed from the aortic valve annulus to the diaphragm. Therefore, delivery from the femoral artery to the iliac artery cannot be simulated using the current model. Second, the model does not include an actual vessel with three layers. The pressure slightly expanded the model; therefore, endoleak evaluation was impossible using the current model. Finally, performing simulations of individual cases using this model is cost-intensive. We are developing a pressurized acrylic box with an iliac access model for advanced training, including puncture and delivery, and a simple, cost-effective simulator for repeated training.

## CONCLUSION

The 3D aortic model was a useful tool to simulate both open and endovascular surgical simulation and training as hybrid aortic surgery for TAAD and TBAD.

## Data Availability

The article’s data will be shared upon reasonable request from the corresponding authors.

## Notes

Presented at the 38th European Association for Cardio-Thoracic Surgery, Lisbon, Portugal, October 11, 2024.
